# Evolution of infant feeding practices in children from 9 to 24 months, considering complementary feeding indicators and food processing: Results from the Brazilian cohort of the MAL‐ED study

**DOI:** 10.1111/mcn.13413

**Published:** 2022-08-15

**Authors:** Eva Débora de Oliveira Andrade, Amanda de Sousa Rebouças, José Q. Filho, Ramya Ambikapathi, Laura E. Caulfield, Aldo Ângelo Moreira Lima, Bruna Leal Lima Maciel

**Affiliations:** ^1^ Graduate Progam in Nutrition Federal University of Rio Grande do Norte Natal Brazil; ^2^ Department of Nutrition Federal University of Rio Grande do Norte Natal Brazil; ^3^ Institute of Biomedicine for Brazilian Semiarid, Faculty of Medicine Federal University of Ceará Fortaleza Brazil; ^4^ Department of Public Health Purdue University West Lafayette Indiana USA; ^5^ Department of International Health, Center for Human Nutrition The Johns Hopkins Bloomberg School of Public Health Baltimore Maryland USA

**Keywords:** breastfeeding, children, complementary feeding, ultra‐processed food

## Abstract

Infant feeding practices impact children's nutritional and health status, influencing growth and development. This study aimed to analyse the evolution of infant feeding practices from 9 to 24 months of age, considering infant and young child feeding (IYCF) indicators and food processing. The infant feeding practices in children from the Brazilian site of the MAL‐ED study were evaluated at 9 (*n* = 193), 15 (*n* = 182) and 24 months (*n* = 164) using 24‐h dietary recalls. IYCF indicators were evaluated, and the extent of food processing was evaluated, using the NOVA classification. Breastfeeding declined significantly over time, from 77.6% at 9 months to 45.1% at 24 months. Although dietary diversity did not significantly change during the study period (80.5% at 24 months), the minimum acceptable diet significantly increased from 67.9% to 76.1% at 24 months (*p* < 0.0005). All the studied children consumed sweetened beverages from 9 months. Unhealthy food consumption and zero vegetable or fruit consumption significantly increased over time (*p* < 0.0005). Unprocessed food consumption decreased from 9 to 24 months of age (*p* < 0.0005), while ultra‐processed food consumption increased (*p* < 0.0005) during the study period. Logistic regressions showed that, at 9 months, breastfed children presented a lower risk for ultra‐processed food consumption (odds ratio [OR] = 0.31; 95% confidence interval [CI] = 0.13–0.77); and children reaching the minimum acceptable diet presented more risk for ultra‐processed food consumption (OR = 2.31; 95% CI = 1.01–5.27). In conclusion, data showed a reduction in the quality of infant feeding practices over the first 2 years of life, with a decrease in breastfeeding and an increase in the consumption of unhealthy and ultra‐processed foods.

## Key messages

- Data showed a reduction in the quality of infant feeding practices over the first 2 years of life.
- Breastfeeding decreased over the studied period. There was an increase in the consumption of ultra‐processed and unhealthy foods, which occurred concomitantly to the increase of no consumption of fruits/vegetables.
- Breastfeeding was an important protective factor against a greater consumption of ultra‐processed food.
- National nutrition policy should consider promoting breastfeeding and increasing fresh and minimally processed food consumption in children under 2 years.

## Graphical abstract

The article aimed to analyse the evolution of infant feeding practices from 9 to 24 months of age in children from the Brazilian cohort of the MAL‐ED study. The study showed a reduction in the quality of infant feeding practices over the first 2 years of life, with a decrease in breastfeeding and an increase in the consumption of unhealthy and ultra‐processed foods.

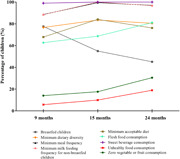

## 1 INTRODUCTION

1.1

Infant feeding practices, including breastfeeding and complementary feeding, influence children's nutritional and health status, generating impacts on growth and development (World Health Organization, 2008). Inadequate infant feeding practices in the first years of life increase the risk of malnutrition and infections, which, therefore, negatively affect growth and development (Guerrant et al., 2008; World Health Organization, 2001, 2008). Inappropriate practices can also increase the risk of overweight/obesity in childhood, increasing the risk for related comorbidities (Gonçalves et al., 2019; Schneider et al., 2020; Victora et al., 2008).

According to the World Health Organization—WHO (World Health Organization, 2000), breastfeeding should be exclusively offered to children until 6 months. From that age onwards, solid foods and beverages should be provided, in addition to breast milk.

The WHO/UNICEF Technical expert advisory group on nutrition monitoring (TEAM) created a set of indicators, recently updated (World Health Organization & the United Nations Children's Fund UNICEF, 2021), to assess infant and young child feeding (IYCF) practices. Studies should prioritize these indicators to provide concise information on health status and trends, including responses at national and global levels (World Health Organization, 2008; World Health Organization & the United Nations Children's Fund UNICEF, 2021).

Thus, studies aimed to understand childhood nutrition through the characterization of dietary practices in local communities (Raymond et al., 2017); Schneider et al. (2020) described the association of complementary feeding during the first year of life with the risk of overweight at 24 months of age, using the birth cohorts of Pelotas (Brazil) from 2004 and 2015. In both cohorts, most of the children received complementary feeding earlier than recommended, and this early introduction was associated with a higher risk for overweight at the age of 24 months. Others reported on select IYCF indicators (Owais et al., 2016) and their relationship with nutritional status (Campbell et al., 2017; Ersino et al., 2016; Owais et al., 2016).

In Brazil, a large national cross‐sectional study was conducted from February 2019 to March 2002, called the ENANI‐2019 study, using a probabilistic sample of children distributed in 123 municipalities in the 26 states of the Federation and the Federal District (*n* = 14,558 children). The study evaluated dietary practices in children under 2 years of age and found that exclusive breastfeeding was 45.8%, continued breastfeeding was 43.6%, and complementary feeding from 6 to 8 months was 86.3% (UFRJ. Federal University of Rio de Janeiro, 2021a, 2021b). Other cross‐sectional Brazilian studies also analysed children's eating habits using the IYCF indicators. Most of these studies showed that few children were exclusively breastfed in the first 6 months, receiving an adequate number of meals per day, but with low dietary diversity (Gonsalez et al., 2017; Isabel et al., 2010; Lindsay et al., 2008; Oliveira et al., 2014; Saldan et al., 2015).

Thus, although several studies describe breastfeeding both globally and locally using the IYCF indicators (Horta et al., 2007; World Health Organization, 2000, 2001, 2003, 2006, 2008), the same number of studies have not been done to characterize complementary feeding, with few prospective analyses. This fact can be explained by the logistical difficulty in applying and analysing 24‐h recalls, as the prospective assessment of populations is complex, even for the observation of the IYCF indicators (P. N. Costa et al., 2020).

However, prospective studies are essential for understanding the trends and evolution of dietary practices over the first 2 years of life. Furthermore, they can help plan more assertive public policies to promote child health. In addition to the IYCF indicators for evaluating dietary practices, it is also necessary to recognize that the quality of food offered in complementary food is essential. Thus, the extent of food processing has been used to characterize the quality of food consumed in some studies. The majority of these studies were cross‐sectionally designed, analysing children older than 2 years, demonstrating a significant presence of food with a high degree of processing in the diet of the studied populations (Cornwell et al., 2018; C. S. Costa et al., 2019; Filgueiras et al., 2019; Rauber et al., 2015; Spaniol et al., 2020).

Some Brazilian researchers have performed an analysis of complementary feeding, considering the extent of food processing (Fonseca et al., 2019; Giesta et al., 2019; Passanha et al., 2021; Ribas et al., 2021; Santos et al., 2018). They concluded that the feeding practices of children between 4 and 24 months were inappropriate, with high ultra‐processed food consumption. However, they presented the limitations of performing a cross‐sectional analysis (Fonseca et al., 2019; Passanha et al., 2021; Santos et al., 2018) or retrospective analysis of hospitalized children (Giesta et al., 2019; Ribas et al., 2021).

Therefore, although studies have also found data supporting the hypothesis that the extent of processing in food is negatively associated with nutritional status and health outcomes in childhood (Cornwell et al., 2018; C. S. Costa et al., 2019; Filgueiras et al., 2019; Fonseca, Ribeiro, Andreoli, 2019; Rauber et al., 2015; Spaniol et al., 2020), food processing in complementary feeding has not been extensively studied.

Rocha et al. (2021) evaluated commercial baby food availability and child nutritional status in Natal, Brazil. They found that the food was predominantly ultra‐processed (79%); only 4.2% was minimally processed, with similar proportions of ultra‐processed food found in high‐ and low‐income areas. Hence, it is essential to evaluate the quality of food consumed, considering its processing extent in complementary feeding.

Among studies that allow a prospective analysis of infant feeding practices, there is the birth cohort MAL‐ED study, which aimed to improve the understanding of the complex interrelationships between enteropathogenic infections, food intake, nutritional status, intestinal physiology, cognitive development, immune function, growth and development (Miller et al., 2014). Using the MAL‐ED, Maciel et al. (2018) described infant feeding practices and the determinants of early complementary feeding in Brazilian children from birth to 8 months of age. Here we extend the characterization of this population, using the IYCF indicators and food processing, to better understand the quality of complementary feeding. The hypothesis under study is that infant feeding practices worsen over time, with breastfeeding reduction and increased consumption of less healthy ultra‐processed food.

## 2 METHODOLOGY

### 2.1 Study population

The present work is part of the birth cohort, The Etiology, Risk Factors and Interactions of Enteric Infections and Malnutrition and the Consequences for Child Health and Development, known as the MAL‐ED study (Miller et al., 2014) with data referring to the Brazilian site of the cohort (Lima et al., 2014). The community under investigation is a slum, known as *Parque universitário*, located in Fortaleza, capital of *Ceará* state. The population of this community is 42,494 thousand inhabitants, according to the latest Brazilian demographic census (IBGE, 2003). A census of the local community was performed to obtain an assessment of the number of women of reproductive age and the number of children <5 years of age. With these data, a catchment area was identified for which it was estimated that >200 infants (the target number of children to be enrolled per site in the MAL‐ED study) would be born within the 2‐year enrolment period (Miller et al., 2014).

The criteria for inclusion in the study were: healthy newborn child, up to 17 days after birth; birth weight greater than 1500 g; child from a family that intended to remain in the study area for the next 6 months; no other children from the same family in the study and a mother at the age of 16 or over. Children were excluded from the study if they were premature, had congenital diseases, serious illnesses that required hospitalization or any other condition that was serious or chronic, such as kidney disease, chronic heart failure or severe liver disease (Miller et al., 2014). The study obtained ethical approval and the research participants were included in the study after the informed consent of the mother or caregiver (Miller et al., 2014).

The open birth cohort enrolled in November 2010 was followed up until February 2017, with children being monitored from birth to 5 years. The enrolment was performed within the first 2 years of the cohort. and follow‐up was performed by local health community agents in the children's households for dietary and anthropometry assessments. In the Brazilian MAL‐ED site, 244 children were included at birth, 11 children left the study due to a change of location or abandonment of the study, totalling 233 children in the cohort (Lima et al., 2014). As described by Lima et al. (2014), most of the households (87%) had access to clean water, 98% had electricity and 69% had access to improved toilet/sanitation. Most childbirths occurred at the hospital, and the under‐5 mortality rate was 20 per 1000 live births.

**Figure 1:**
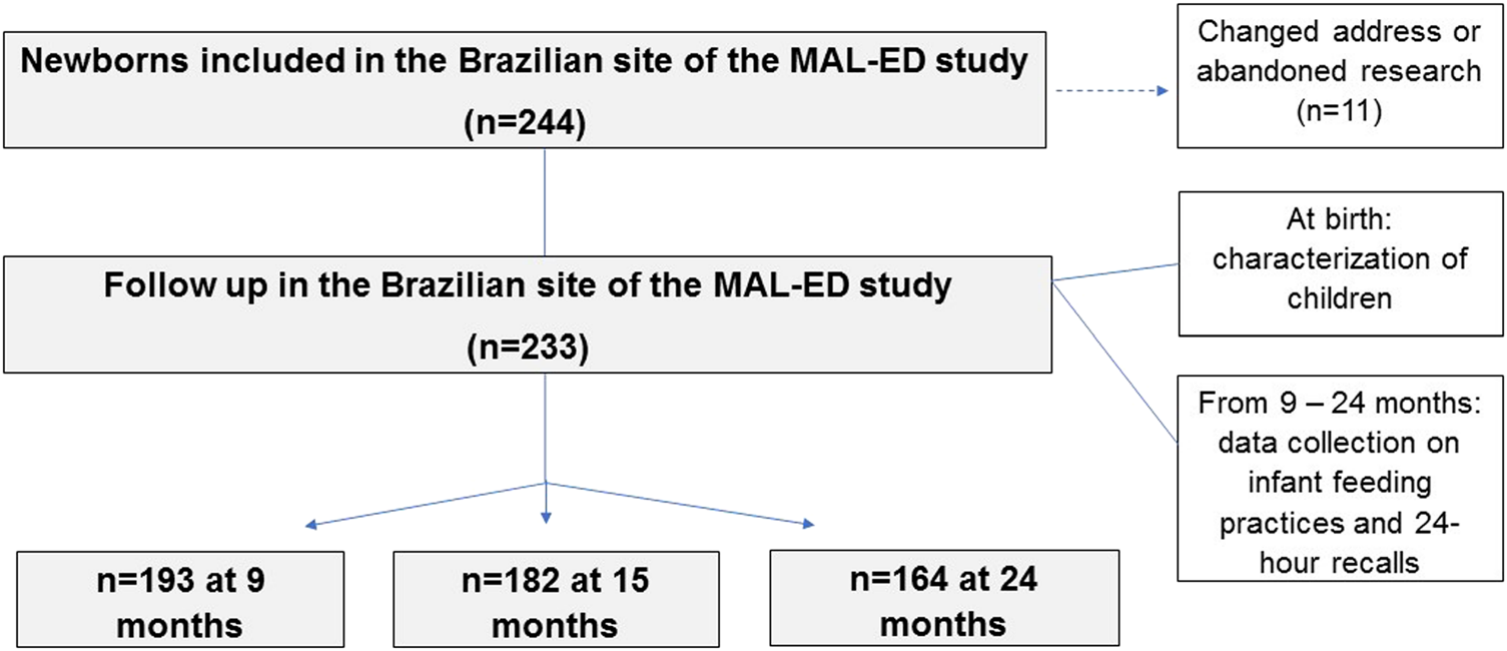
Inclusions, losses and data collection flowchart of the studied population

The present work used data from birth, 9, 15 and 24 months of age. Birth data were used to characterize the studied children. The follow‐up of 9 months was evaluated because it was the first month of 24‐h recall registration in the MAL‐ED study, as discussed by Caulfield et al. (2014). Data from 15 months were used because they represented a middle point from 6 to 24 months with a good sample size in the study. In the study, 193 children at 9 months, 182 children at 15 months and 164 children at 24 months completed the 24‐h recalls (Figure 1). Considering a total sample of 233, losses in the dietary follow‐up were 17% at 9 months, 22% at 15 months and 29% at 24 months. Power analysis was performed a posteriori, considering our smallest sample size (*n* = 164, at 24 months), an effect size of 0.3, a given *α* of 0.05 and two degrees of freedom. Power was 94%, and the critical *X* ^2^ = 5.99.

### 2.2 Food intake

In the MAL‐ED study, food intake was verified monthly from 9 to 24 months of age through 24‐h recalls. For data collection, the caregiver was asked to remember the food intake in the last 24h and all foods and meals given to the children were registered, using a common form across the MAL‐ED sites (Caulfield et al., 2014). These data were collected by trained field investigators, including details on ingredients and preparation steps, mainly for local preparations. Food composition analysis was performed using the Brazilian food composition tables (TACO) and the Brazilian Institute of Geography and Statistics (IBGE), food labels and the USDA table by staff trained at Johns Hopkins University, Baltimore, MD (Caulfield et al., 2014).

In the present research, recalls from 9, 15 and 24 months of age were used. Based on these data, infant feeding practices and food processing levels were evaluated.

### 2.3 Infant feeding practices

The infant feeding practices were assessed from the children's 24‐h recalls, using the following IYCF indicators (World Health Organization & the United Nations Children's Fund UNICEF, 2021): (1) breastfeeding; (2) minimum dietary diversity; (3) minimum meal frequency; (4) minimum milk feeding frequency for non‐breastfed children; (5) minimum acceptable diet; (6) egg and/or flesh food consumption; (7) sweet beverage consumption; (8) unhealthy food consumption and (9) zero vegetable or fruit consumption.

Breastfeeding was verified through monthly interviews for the 24‐h recalls. Minimum dietary diversity was considered to be met when the child received food from five or more of the following groups per day: (1) breast milk; (2) grains, roots and tubers; (3) pulses (beans, peas, lentils), nuts and seeds; (4) Dairy products (milk, infant formula, yoghurt, cheese); (5) flesh foods (meat, fish, poultry, organ meats); 6) eggs; (7) vitamin A‐rich fruits and vegetables and (8) other fruits and vegetables (World Health Organization & the United Nations Children's Fund UNICEF, 2021).

In the present study, the analysis of the 24‐h recalls allowed the visualization of the frequent consumption of sugar, oils and fats. Therefore, these food groups were also included in the studied food groups, but they were not considered in the calculation of minimum dietary diversity.

The minimum meal frequency was assessed as adequate when three feedings of solid, semi‐solid or soft foods were given for breastfed children aged 9–23 months, and four feedings of solid, semi‐solid or soft foods or milk feeds for non‐breastfed children aged 6–23 months whereby at least one of the four feeds was a solid, semi‐solid or soft feed (World Health Organization & the United Nations Children's Fund UNICEF, 2021).

For non‐breastfed children, it was observed if they received milk or dairy products, liquid dairy products, such as infant formula, cow's milk or other animal milk and dairy products at least twice a day, which was considered adequate (World Health Organization & the United Nations Children's Fund UNICEF, 2021).

The minimum acceptable diet was considered met when breastfed children met the criteria of minimum dietary diversity and minimum meal frequency. For non‐breastfed children, besides meeting the criteria of minimum dietary diversity and minimum meal frequency, they should have received at least two milk feeds to meet the minimum acceptable diet (World Health Organization & the United Nations Children's Fund UNICEF, 2021).

Unhealthy foods (sentinel foods) were considered commercially prepared food products that are often energy‐dense, nutrient‐poor, and rich in salt, sugar and saturated and/or trans fatty acids, such as candies, chocolates, cookies, salty biscuits, chips, industrialized popcorn and instant noodles. For this indicator, we calculated the percentage of children who consumed at least one unhealthy food, such as candies, chocolate, chips, French fries, cakes and cookies (World Health Organization & the United Nations Children's Fund UNICEF, 2021).

Zero consumption of vegetables and fruits was the total number of children who did not consume any vegetables or fruits during the previous day (World Health Organization & the United Nations Children's Fund UNICEF, 2021).

### 2.4 Food processing

The foods and beverages consumed were evaluated according to the extent of processing, based on Monteiro et al. (2010) NOVA classification. Examples of foods observed in 24‐h recalls of the studied children according to their extent of processing are shown in Table 1. In the present study, fresh and minimally processed food were considered separately as the 24‐h recalls allowed this differentiation, and this proved important when considering the food groups. The food groups recommended by the WHO for the calculation of minimum dietary diversity were considered to assess the extent of food processing.

**Table 1:** Examples of foods observed in 24‐h recalls of the studied children according to the extent of processing

Extent of food processing	Examples of foods
Unprocessed	Fresh fruits and vegetables.
Minimally processed	Rice, beans, powdered milk (without stabilizers), spaghetti, chilled meat and corn cuscus flakes.
Processed	Foods made by adding salt, oil, sugar or other substances from unprocessed or minimally processed foods: tomato extract or concentrates (with salt or sugar); fruits in syrup and candied fruits; dried meat and bacon; canned sardines and tuna; bread made from wheat, yeast, water and salt.
Ultra‐processed	Foods made of formulations of ingredients, mostly of exclusive industrial use, typically created by a series of industrial techniques and processes: industrialized cookies, ice cream, chocolates, candies and sweets in general. Industrialized chips, popcorn, salty biscuits, cereal bars, soups and instant noodles. Industrialized spices and sauces, sodas, sweetened flavoured yoghurts and sweetened flavoured milk drinks. Frozen, ready‐to‐heat industrialized products, such as pasta dishes and pizzas. Industrialized hamburgers, fish or chicken nuggets and sausages. Sliced bread, bread for hamburgers or hot dogs, sweet buns and other baked products with ingredients, such as hydrogenated vegetable fat, whey, emulsifiers and other additives.

The analysis of the extent of food processing in the studied food groups revealed that: (1) the legumes consumed were all minimally processed; (2) there was no significant consumption of nuts, seeds or eggs in the studied population; (3) Fruit and vegetables consumed were classified as fresh, unprocessed food. Thus, the food groups ‘grains, roots and tubers’, ‘dairy products’ and ‘flesh foods’ were presented considering the extent of processing.

The number and frequency of food consumed according to the level of processing were evaluated. This was done by counting the different foods consumed daily by the child, classifying each food according to processing (e.g., the child consumed seven different kinds of food from a given group throughout the day, three of these belonged to the fresh group, two to the minimally processed group and two to the ultra‐processed group). The food consumption frequency considered the number of times the child received the same food a day.

### 2.5 Statistical analysis

Quantitative variables were tested for normality using the Kolmogorov−Smirnov test. Variables without normal distribution were presented as median (Q1–Q3) and variables with normal distribution as mean (standard deviations). Categorical variables were presented as absolute and relative frequencies, using the *χ* ^2^ test. The medians of the number and frequency of consumption of the food groups were tested using the Kruskal−Wallis test.

Factors associated with high consumption of ultra‐processed food were analysed in logistic regression models. First, the number of ultra‐processed food consumed at each age (9, 15 and 24 months) was divided into quartiles. Children were categorized in relation to consumption ≥ than the Q3 (yes = 1 or no = 0). Then, logistic regression models were calculated, primarily in bivariate analysis, exploring the effect of a single variable on the consumption of ultra‐processed foods greater than Q3 (yes = 1 or no = 0) at 9, 15 and 24 months, with the unadjusted odds ratio (OR) and their respective 95% confidence intervals (95% CI) demonstrated. The logistic regression models were calculated for each studied period (9, 15 and 24 months), considering the consumption of ultra‐processed food higher than Q3 (yes = 1 or no = 0) as a dependent variable. The adjustment of the three final models was evaluated using the Omnibus test, with *p* values less than 0.05, and the Hosmer and Lemeshow test, considering *p* values greater than 0.05. Maternal education at birth, income, breastfeeding, minimum acceptable diet and the number of unprocessed food consumed per day entered the final models as independent variables. The adjusted odds ratios (AOR) and their respective 95% CI were presented in the models. The analysis was performed using the SPSS statistical program, version 23 (IBM).

## 3 RESULTS

Table 2 shows the general characteristics of the studied children: 51.5% were male, the mean birth weight was 3360 (499) g, and the median maternal education was 7.0 (6.0–8.0) years. Household income increased during the study period from $236 (229–307) at 6 months to $308 (276–621) at 24 months.

**Table 2:** Sex, birth weight, maternal education and family income of the Brazilian children from the MAL‐ED cohort (*n* = 233)

Variables	Total
Male child (birth), *n* (%)	120 (51.5)
Birth weight (g), mean (SD)	3360 (499)
Maternal education (years), median (Q1–Q3)	7.0 (6.0–8.0)
Family income ($), median (Q1 – Q3)	
At 6 months of age	236 (229–307)
At 12 months of age	272 (229–339)
At 24 months of age	308 (276–361)

**Figure 2:**
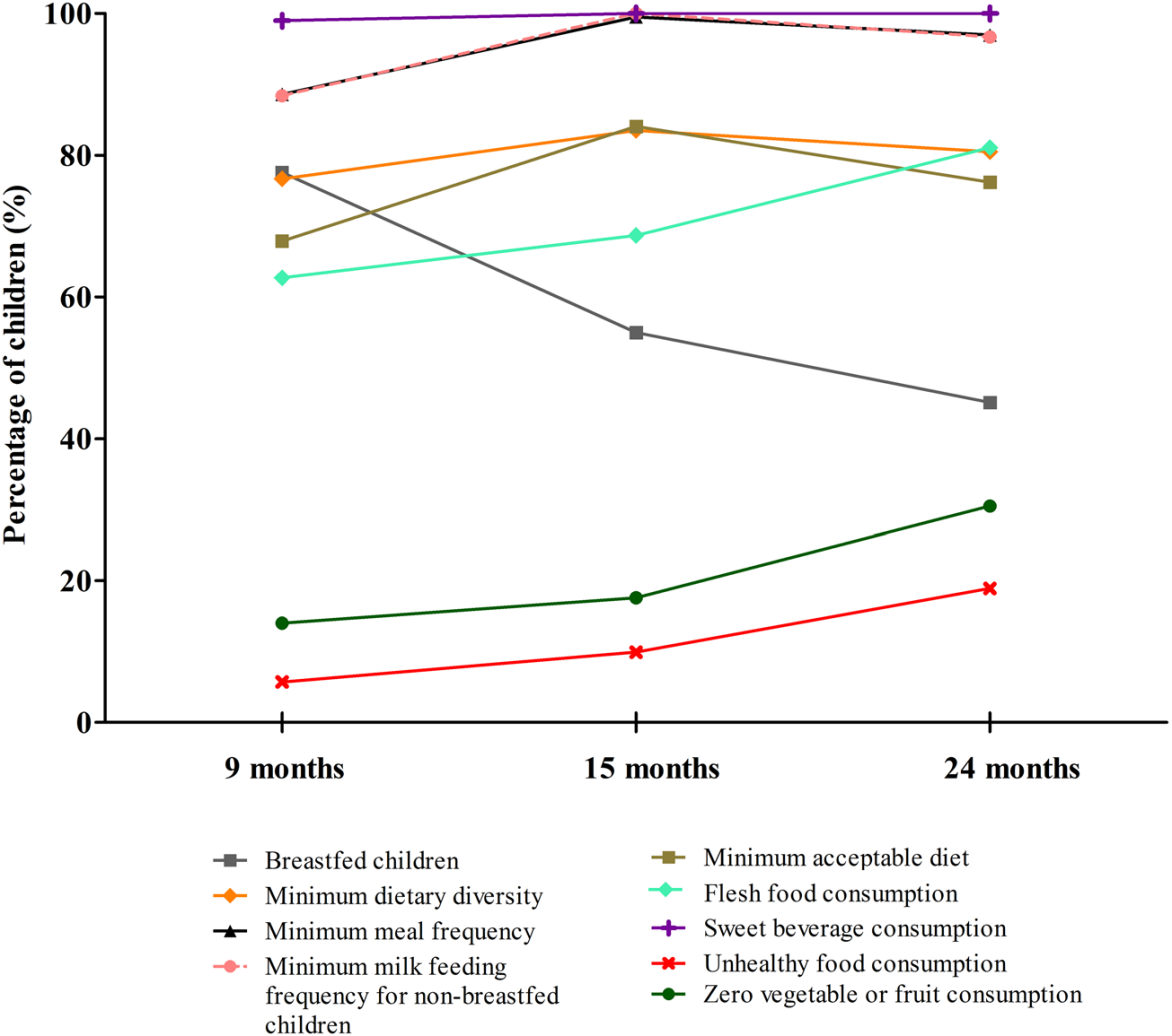
Infant feeding practices in the studied children at 9 (*n* = 193), 15 (*n* = 182) and 24 (*n* = 164) months old, assessed using the following IYCF indicators: breastfed children; minimum dietary diversity; minimum meal frequency; minimum milk feeding frequency for non‐breastfed children; minimum acceptable diet; flesh food consumption; sweet beverage consumption; unhealthy food consumption; zero vegetable or fruit consumption. IYCF, infant and young child feeding.

The prevalence of breastfeeding declined over time, from 77.6% at 9 months to 55.0% at 15 months and 45.1% at 24 months (*χ* ^2^, *p* < 0.0005; Figure 2). Minimum dietary diversity did not increase significantly during the study period (*χ* ^2^, *p* = 0.251) and was 76.7% at 9 months, 83.5% at 15 months and 80.1% at 24 months (Figure 2). Minimum meal frequency significantly increased during the study period (*χ* ^2^, *p* < 0.0005): from 88.6% at 9 months, to 99.5% at 15 months and 97% at 24 months (Figure 2). The frequency of milk consumption among non‐breastfed children also significantly increased over time (*χ* ^2^, *p* = 0.005) and was 96.7% at 24 months (Figure 2). The minimum acceptable diet significantly increased over the study period (*χ* ^2^, *p* = 0.001) and was 67.9% at 9 months, 84.1% at 15 months and 76.1% at 24 months (Figure 2).

Flesh food consumption significantly increased over the months (*χ* ^2^, *p* = 0.001) to 81.1% at 24 months (Figure 2). Sweetened beverages were consumed by all children from 15 months (*χ* ^2^, *p* = 0.165; Figure 2). These were mostly industrialized ultra‐processed flavoured liquid or powdered juices. Unhealthy food consumption significantly increased (*χ* ^2^, *p* < 0.0005) from 5.7% at 9 months, to 9.9% at 15 months and 18.9% at 24 months (Figure 2). Zero vegetable or fruit consumption also significantly increased (*χ* ^2^, *p* < 0.0005) from 14.0% at 9 months, 17.6% at 15 months and 30.5% at 24 months (Figure 2).

The number and frequency of foods consumed were assessed by food groups, considering those recommended by the WHO to estimate the minimum dietary diversity (Table 3). The number and frequency of ‘Grains, roots and tubers’, ‘pulses, nuts and seeds’, ‘sugar, fats and oils’ consumed per day increased during the study period (Kruskal−Wallis, *p* < 0.0005). The number and frequency of ‘Vitamin A‐rich fruits and vegetables’ and ‘other fruits and vegetables’ consumed tended to decrease over the studied period (Kruskal−Wallis, *p* < 0.0005), and was only 1.0 (0.0–1.0) per day at 24 months.

**Table 3:** Number and frequency of food consumed per day by the studied children at 9, 15 and 24 months of age, considering food groups

Food group	Number of foods consumed per day			*p* value, Kruskal−Wallis test
	9 months (*n* = 193)	15 months (*n* = 182)	24 months (*n* = 164)	
	Median (Q1–Q3)	Median (Q1–Q3)	Median (Q1–Q3)	
Grains, roots and tubers	4.0 (3.0–4.0)	4.0 (3.0–5.0)	4.0 (4.0–4.0)	<0.0005
Pulses (beans, peas lentils), nuts and seeds	0.0 (0.0–0.0)	0.0 (0.0–1.0)	1.0 (0.0–1.0)	<0.0005
Dairy products	1.0 (1.0–2.0)	1.0 (1.0–2.0)	2.0 (1.0–2.0)	0.002
Flesh foods	1.0 (0.0–1.0)	1.0 (0.0–1.0)	1.0 (1.0–1.0)	0.525
Eggs	0.0 (0.0 – 0.0)	0.0 (0.0 – 0.0)	0.0 (0.0 – 0.0)	*
Vitamin A‐rich fruits and vegetables	1.0 (0.0–2.0)	1.0 (0.0–1.0)	1.0 (0.0–1.0)	<0.0005
Other fruits and vegetables	1.0 (1.0–2.0)	1.0 (0.0–3.0)	1.0 (0.0–1.0)	<0.0005
Sugar, oils and fats	1.0 (1.0–2.0)	2.0 (1.0–2.0)	2.0 (1.0–2.0)	<0.0005

	Frequency of foods consumed per day			
	9 months (*n* = 193)	15 months (*n* = 182)	24 months (*n* = 164)	
Food group	Median (Q1–Q3)	Median (Q1–Q3)	Median (Q1–Q3)	*p* value, Kruskal−Wallis test
Grains, roots and tubers	7.0 (5.0–9.0)	8.0 (6.0–9.0)	8.0 (6.0–9.0)	0.011
Pulses (beans, peas lentils), nuts and seeds	0.0 (0.0–0.0)	0.0 (0.0–1.0)	1.0 (0.0–1.0)	<0.0005
Dairy products	3.0 (2.0–4.0)	3.5 (2.0–4.0)	3.0 (3.0–4.0)	0.992
Flesh foods	1.0 (0.0–1.0)	1.0 (0.0–1.0)	2.0 (1.0–2.0)	<0.0005
Eggs	0.0 (0.0–0.0)	0.0 (0.0–0.0)	0.0 (0.0–0.0)	*
Vitamin A‐rich fruits and vegetables	1.0 (0.0–2.0)	1.0 (0.0–1.0)	1.0 (0.0–1.0)	<0.0005
Other fruits and vegetables	1.0 (1.0–2.0)	1.0 (0.0–3.0)	1.0 (0.0–1.0)	<0.0005
Sugar, oils and fats	4.0 (3.0–5.0)	4.0 (3.0–5.0)	5.0 (4.0–6.0)	<0.0005

* Not calculated, no significant consumption and variation in the population studied.

Foods consumed within each group shown in Table 3 were also analysed, considering food processing, with the number and frequency of consumption evaluated per day (Table 5). The number of unprocessed foods consumed decreased during the study period: from a median of 3.0 (1.0–4.0) at 9 months to 2.0 (1.0–2.0) at 24 months (Kruskal−Wallis, *p* < 0.0005, Table 5). The frequency of unprocessed food consumption also reduced over the study period, from a median of 3.0 (2.0–5.0) per day at 9 months, to 2.0 (1.0−2.0) at 24 months (Kruskal−Wallis, *p* < 0.0005, Table 5). On the other hand, ultra‐processed food consumption increased significantly over time: from 2.0 (1.0−2.0) foods, 3.0 (3.0−4.0) times a day at 9 months to 2.0 (2.0−2.0) foods, 4.0 (3.0−4.0) times a day at 24 months (Kruskal−Wallis, *p* < 0.0005, Table 5).

Ultra‐processed food was consumed by 96.4% of children at 9 months, 99.5% at 15 months and 99.4% at 24 months. Regarding ‘grains, roots and tubers’, there was a higher consumption of minimally processed and ultra‐processed food, with a reduction in the consumption of unprocessed and an increase in the consumption of ultra‐processed foods over the studied period (Kruskal−Wallis, *p* < 0.0005, Table 5). The most frequently consumed foods in this group were flours for porridge, which were all sweetened and sometimes flavoured (ultra‐processed food), spaghetti (minimally processed food) and rice (minimally processed food) (Table 4).

**Table 4:** Most consumed food, considering food groups in the studied children at 9, 15 and 24 months

Consumed food	9 months, *n* (%)	15 months, *n* (%)	24 months, *n* (%)	*p* value, *χ* ^2^ test
Grains, roots and tubers				
Bread	0 (0.0)	7 (3.9)	5 (3.1)	0.029
English potato	51 (26)	46 (25.2)	39 (23.8)	0.832
Flours for porridge	168 (87.0)	180 (98.9)	115 (71.9)	<0.0005
Rice	60 (31.1)	144 (79.1)	130 (79.3)	<0.0005
Spaghetti	36 (18.7)	108 (59.3)	115 (70.1)	<0.0005
Pulses				
Beans	24 (12.4)	36 (20)	28 (17.1)	0.150
Dairy products				
Chocolate milk	1 (0.5)	10 (5.5)	21 (12.8)	<0.0005
Yoghurt (flavoured/sweetened)	48 (24.8)	76 (41.8)	109 (66.5)	<0.0005
Powdered milk	125 (64.8)	150 (82.4)	135 (82.3)	<0.0005
UHT milk	23 (11.9)	31 (17.0)	22 (13.4)	0.347
Flesh food				
Red meat	31 (16.1)	43 (23.6)	43 (26.2)	0.050
Chicken	53 (27.5)	65 (35.7)	71 (43.3)	0.007
Vitamin A‐rich fruits and vegetables				
Carrots	26 (13.5)	20 (11.0)	18 (11.0)	0.693
Other fruit and vegetables				
Banana	62 (32.1)	51 (28)	38 (23.2)	0.172
Beet	19 (9.8)	14 (7.7)	14 (8.5)	0.758
Orange	23 (11.9)	24 (13.2)	27 (16.5)	0.446
Apple	35 (18.1)	29 (15.9)	26 (15.9)	0.800
Culinary ingredients				
Sugar	182 (94.3)	177 (97.2)	152 (92.7)	0.149
Margarine/butter	12 (6.2)	21 (11.5)	32 (19.5)	0.001
Vegetable oils	64 (33.2)	72 (39.6)	81 (49.4)	0.008
Sentinel foods				
Candies	5 (2.6)	12 (6.6)	22 (13.4)	<0.0005
Chocolates	3 (1.6)	9 (4.9)	14 (8.5)	0.009
Cookies/salty biscuits	12 (6.2)	90 (49.5)	106 (64.6)	<0.0005
Chips/industrialized popcorn	1 (0.5)	13 (7.1)	25 (15.2)	<0.0005
Instant noodles	6 (3.1)	21 (11.5)	26 (15.9)	<0.0005

The number and frequency of ultra‐processed ‘dairy products’ consumed increased during the study period: from 0.0 (0.0−1.0) at 9 months to 1.0 (1.0−1.0) at 24 months (Kruskal−Wallis, *p* = 0.007, Table 5). The most consumed foods in this group were powdered milk (minimally processed food) and yoghurts, which were all flavoured and sweetened (ultra‐processed food) (Table 4). Most ‘flesh foods’ were minimally processed (Table 5), and were most often chicken or beef (Table 4).

**Table 5:** Number and frequency of food consumed/day by the studied children, considering food processing in different food groups, at 9, 15 and 24 months

Food groups	Number of foods consumed per day			*p* value, Kruskal–Wallis test
	9 months (*n* = 193)	15 months (*n* = 182)	24 months (*n* = 164)	
	Median (Q1–Q3)	Median (Q1–Q3)	Median (Q1–Q3)	
Total of food consumed				
Unprocessed food	3.0 (1.0–4.0)	2.0 (1.0–5.0)	2.0 (1.0–2.0)	<0.0005
Minimally processed food	5.0 (3.0–5.0)	5.0 (5.0–5.0)	5.0 (5.0–5.0)	<0.0005
Processed food	0.0 (0.0–0.0)	0.0 (0.0–0.0)	0.0 (0.0–0.0)	*
Ultra‐processed food	2.0 (1.0–2.0)	2.0 (2.0–2.0)	2.0 (2.0–2.0)	<0.0005
Grains, roots and tubers				
Unprocessed food	1.0 (0.0–1.0)	0.0 (0.0–1.0)	0.0 (0.0–0.0)	<0.0005
Minimally processed food	2.0 (0.0–2.0)	2.0 (1.0–2.0)	1.0 (1.0–1.0)	<0.0005
Processed food	0.0 (0.0–0.0)	0.0 (0.0–0.0)	0.0 (0.0–0.0)	*
Ultra‐processed food	1.0 (1.0–1.0)	2.0 (1.0–2.0)	2.0 (2.0–2.0)	<0.0005
Dairy products				
Unprocessed food	—	—	—	—
Minimally processed food	1.0 (1.0–1.0)	1.0 (1.0–1.0)	1.0 (1.0–1.0)	0.007
Processed food	0.0 (0.0–0.0)	0.0 (0.0–0.0)	0.0 (0.0–0.0)	*
Ultra‐processed food	0.0 (0.0–1.0)	0.0 (0.0–1.0)	1.0 (1.0–1.0)	<0.0005
Flesh foods				
Unprocessed food	—	—	—	—
Minimally processed food	1.0 (0.0–1.0)	1.0 (0.0–1.0)	1.0 (0.0–1.0)	0.166
Processed food	0.0 (0.0–0.0)	0.0 (0.0–0.0)	0.0 (0.0–0.0)	*
Ultra‐processed food	0.0 (0.0–0.0)	0.0 (0.0–0.0)	0.0 (0.0–0.0)	*

	Frequency of foods consumed per day			
	9 months (*n* = 193)	15 months (*n* = 182)	24 months (*n* = 164)	
Food groups	Median (Q1–Q3)	Median (Q1–Q3)	Median (Q1– Q3)	*p* value, Kruskal–Wallis test
Total of food consumed				
Unprocessed food	3.0 (2.0–5.0)	3.0 (1.0–3.0)	2.0 (1.0–2.0)	<0.0005
Minimally processed food	10.0 (6.0–10.0)	10.0 (6.0–10.0)	7.0 (6.0–10.0)	0.308
Processed food	0.0 (0.0–0.0)	0.0 (0.0–0.0)	0.0 (0.0–0.0)	*
Ultra‐processed food	3.0 (3.0–4.0)	4.0 (3.0–4.0)	4.0 (3.0–4.0)	<0.0005
Grains, roots and tubers				
Unprocessed food	1.0 (0.0–1.0)	0.0 (0.0–1.0)	0.0 (0.0–0.0)	<0.0005
Minimally processed food	2.0 (0.0–2.0)	2.0 (1.0–2.0)	1.0 (1.0–2.0)	<0.0005
Processed food	0.0 (0.0–0.0)	0.0 (0.0–0.0)	0.0 (0.0–0.0)	*
Ultra‐processed food	3.0 (3.0–4.0)	4.0 (4.0–4.0)	4.0 (4.0–4.0)	<0.0005
Dairy products				
Unprocessed food	—	—	—	—
Minimally processed food	3.0 (2.0–4.0)	3.0 (2.0–4.0)	3.0 (2.0–4.0)	0.984
Processed food	0.0 (0.0–0.0)	0.0 (0.0–0.0)	0.0 (0.0–0.0)	*
Ultra‐processed food	0.0 (0.0–1.0)	0.0 (0.0–1.0)	1.0 (1.0–1.0)	<0.0005
Flesh foods				
Unprocessed food	—	—	—	—
Minimally processed food	1.0 (0.0–1.0)	1.0 (0.0–1.0)	1.0 (1.0–1.0)	<0.0005
Processed food	0.0 (0.0–0.0)	0.0 (0.0–0.0)	0.0 (0.0–0.0)	*
Ultra‐processed food	0.0 (0.0–0.0)	0.0 (0.0–0.0)	0.0 (0.0–0.0)	*

* Not perfomed ‒ no variation in data.

For the group of ‘grains, roots and tubers’ (Table 4), there was a significant increase in the percent of children consuming rice, spaghetti and bread during the study period; and in ‘dairy products’ of flavoured/sweetened yoghurts and powdered milk. The most frequently consumed ‘vitamin A‐rich vegetable’ (carrots) and ‘other fruit and vegetables’ (banana, beet, orange and apple) did not change frequency during the study period. Sugar was consumed by almost all the studied children (94.3%) from 9 months, with no significant change during the study period. This sugar was mostly added to powdered milk to prepare porridges, using ultra‐processed sweetened flours. The proportion of children consuming margarine/butter and vegetable oils increased significantly during the study period. Margarine/butter was mostly used in bread or salty biscuits, whereas vegetable oils were used in family preparations, such as soups, fried chicken or meat.

The most consumed sentinel foods (all ultra‐processed foods) were cookies/salty biscuits, instant noodles and chips/industrialized popcorn. The number of children consuming these foods significantly increased over the studied period (*χ* ^2^, *p* < 0.0005) (Table 4).

Among the factors associated with higher consumption of ultra‐processed foods (Table 6), the logistic regressions showed that, at 9 months, children who were breastfed were less likely to have a high consumption of ultra‐processed food (AOR = 0.31; 95% CI = 0.13−0.77), whereas children who reached the minimum acceptable diet were more likely to receive a high amount of ultra‐processed food (AOR = 2.31; 95% CI = 1.01−5.27).

**Table 6:** Logistic regression models for ultra‐processed food consumption at 9, 15 and 24 months and associated factors

Independent variables	Consumption of ultra‐processed food at 9 months ≥ Q3		Consumption of ultra‐processed food at 15 months ≥ Q3		Consumption of ultra‐processed food at 24 months ≥ Q3	
	Non‐adjusted OR (95% IC)	Adjusted OR (95% IC)	Non‐adjusted OR (95% IC)	Adjusted OR (95% IC)	Non‐adjusted OR (95% IC)	Adjusted OR (95% IC)
Maternal education	1.22 (0.89–1.68)	1.15 (0.79–1.67)	1.01 (0.68–1.52)	0.87 (0.54–1.41)	1.22 (0.66–2.24)	0.93 (0.47−1.84)
Income	1.00 (1.00–1.00)	1.00 (0.99−1.00)	1.00 (1.00−1.00)	1.00 (1.00−1.00)	1.00 (1.00−1.01)	1.00 (1.00−1.01)
Breastfeeding						
No	—	—	—	—	—	—
Yes	0.47 (0.21−1.02)	**0.31 (0.13−0.77)**	0.95 (0.43−2.08)	0.83 (0.33−2.08)	0.33 (0.08−1.32)	0.52 (0.12−2.21)
Minimum acceptable diet						
No	—	—	—	—	—	—
Yes	2.00 (1.07−3.73)	**2.31 (1.01−5.27)**	1.80 (0.69−4.69)	1.81 (0.59−5.53)	0.00 (0.00−0.00)	0.00 (0.00−0.00)
Number of unprocessed food consumed/day	1.13 (0.97−1.31)	0.99 (0.82−1.19)	1.09 (0.90−1.32)	1.03 (0.84−1.28)	1.01 (0.70−1.47)	1.02 (0.69−1.52)

*Note*: Unadjusted ORs were calculated using logistic regressions in bivariate analysis, exploring the effect of the independent variable alone on the outcomes studied. Q3 of ultra‐processed food consumed per day was 2, at 9, 15 and 24 months.

Abbreviation: OR, odds ratio.

## 4 DISCUSSION

This study investigated the evolution of infant feeding practices in children from 9 to 24 months, using 24‐h recalls. In addition to using the IYCF indicators, we also observed the quality of consumed food, evaluating its processing. Most of the studied children achieved the minimum dietary diversity, minimum meal frequency and minimum acceptable diet. Gatica‐Domínguez et al. (2021), in low‐ and middle‐income countries, demonstrated that only 21.3%, 56.2% and 10.1% of the 80 countries studied had >50% prevalence for dietary diversity, minimum meal frequency and minimum acceptable diet, respectively. For Latin America and the Caribbean, the same study found 56.5%, 71.5% and 43.7% of minimum dietary diversity, minimum meal frequency and minimum acceptable diet, respectively (Gatica‐Domínguez et al., 2021). The national Brazilian ENANI‐19 study found 57.1% of minimum dietary diversity and 39.2% of minimum meal frequency (UFRJ. Federal University of Rio de Janeiro, 2021b). Therefore, our results might indicate greater diet quality than has been reported in other studies.

There was a significant increase in the consumption of vegetables or fruit and unhealthy foods. Sweetened beverages were consumed by all children from 15 months. Schneider et al. (2020), in their work with children during the first year of life, found that juice was introduced before 3 months in half of the children and before 12 months by 95.5% in 2004 and 87.8% in 2015. In addition, nearly 20.0% of children in 2015 received soft drinks before 12 months of age. Excess consumption of sweetened beverages and ultra‐processed food is related to the risk of overweight, obesity and childhood diabetes (Berkey et al., 2004; Davis et al., 2010). On the other hand, the low consumption of vegetables and fruit is associated with nutritional disorders, especially anaemia, malnutrition and overweight/obesity (World Health Organization & the United Nations Children's Fund UNICEF, 2021).

The widespread consumption of sugar by children from 9 months is similar to that found by Filha et al. (2012), in a study with children from 6 to 35 months in five health units in Aracajú, northeast Brazil. Excess sugar consumption is associated with the presence of cavities in childhood and increases the risk of overweight (Berkey et al., 2004; Davis et al., 2010; Malik & Willett, 2013; Vos et al., 2017). Therefore, the Brazilian Ministry of Health (Brasil, 2019) and the World Health Organization (2018) recommend introducing sugar only after 24 months of age, which was not observed in the studied population.

The prospective analysis of the groups of food consumed showed there was a low consumption of pulses, no consumption of eggs and a tendency towards a reduction in the consumption of fruit and vegetables in general. These types of food help prevent nutritional disorders prevalent in childhood, such as anaemia, malnutrition and overweight/obesity, being sources of nutrients, such as iron, zinc, calcium, vitamin A, vitamin C and folate (Dewey, 2001; World Health Organization, 2003). Thus, their consumption must be promoted in the study population. Santos et al. (2018), in a cross‐sectional study on complementary feeding, in Juiz de Fora, Minas Gerais, also found a low consumption of fruit, vegetables and eggs. However, they observed a high consumption of pulses, with around 78% of children receiving pulses twice a day, different from the results in the present study.

Thus, the analysis of the IYCF indicators indicated an unfavourable evolution of infant feeding practices, with a reduction in breastfeeding and concomitant low consumption of eggs and pulses, a tendency to reduce fruit consumption, early consumption of sugar, sweetened beverages, and an increase in the consumption of unhealthy foods. Therefore, the hypothesis that infant feeding practices present an unfavourable evolution was confirmed in the study. This hypothesis was reinforced when considering the extent of processing. In the studied population, the frequency of consumption of ultra‐processed foods was higher than that of unprocessed and minimally processed foods. In addition, the consumption of unprocessed and minimally processed foods decreased over time, while the consumption of ultra‐processed foods increased over the months studied. Our results agree with data from the Brazilian ENANI‐2019 study. In this national population survey, a high prevalence (80.5%) of consumption of ultra‐processed foods was found in children from 6 to 23 months (UFRJ. Federal University of Rio de Janeiro, 2021b).

Ultra‐processed food consumption is associated with low diet quality (Moubarac et al., 2013; Monteiro et al., 2019), obesity (Cornwell et al., 2018) and non‐communicable chronic diseases (Monteiro et al., 2019; Rauber et al., 2015). Thus, the increased consumption of these kinds of food has become the target of public health nutrition policies worldwide (Brasil, 2014; Monteiro et al., 2019). However, most studies that analysed the consumption of ultra‐processed food in children provided data on older children (Buckley et al., 2019; C. S. Costa et al., 2019; Filgueiras et al., 2019; Hoffman et al., 2020; Mais et al., 2017; Moubarac et al., 2017; Neri et al., 2019; Onita et al., 2021; Ribeiro & de Araújo Pinto, 2021).

In the present study, among the possible factors associated with high consumption of ultra‐processed foods, logistic regressions showed that, at 9 months, breastfed children had a lower likelihood of high consumption of ultra‐processed food. This finding is in line with studies reporting that breast milk consumption is associated with lower consumption of ultra‐processed foods among children between 6 and 12 months (Fonseca et al., 2019; Giesta et al., 2019; Passanha et al., 2021; Spaniol et al., 2020). A possible cause for this lower consumption of ultra‐processed food is that breastfeeding mothers tend to limit their children's exposure to and consumption of unhealthy foods (Dwyer, 2018).

Interestingly, children presenting the minimum acceptable diet were more likely to have a high consumption of ultra‐processed food. This indicator is only met when children reach both the minimum dietary diversity and minimum meal frequency. However, minimum dietary diversity and meal frequency do not consider food processing. Thus, our result can be explained by the fact that children who reached the minimum dietary diversity and the minimum meal frequency relied on ultra‐processed foods and beverages, including sweetened flour for porridges, flavoured/sweetened yoghurt and chocolate milk.

Potential limitations of our study must be mentioned, such as the lack of data about salt used to prepare family meals in the 24‐h recalls. Analysing salt could also measure the quality of children's consumed food, as ultra‐processed foods commonly contain high amounts of salt (Monteiro et al., 2019). Furthermore, Brazilian studies have shown high consumption of salt in adult populations (Pizzol et al., 2019; Rodrigues et al., 2015).

The strengths of the study are the longitudinal design and the description of the number and frequency of food consumed, considering the different levels of processing. Although the data found are not nationally representative and rigorous sampling was not performed, the results may reflect infant feeding practices in slums in northeastern Brazil because the studied population represents a typical slum, as previously discussed by Maciel et al. (2018), where accessibility is challenging because of drug trafficking. In addition, this study is one of the first to collect longitudinal information on infant feeding practices through monthly 24‐h recalls. The collection of longitudinal data allowed a detailed assessment of the evolution of children's eating habits. The food analysis, considering the level of processing, allowed a better evaluation of the quality of infant feeding practices, complementing the IYCF indicators.

In conclusion, data showed a reduction in the quality of infant feeding practices over the first 2 years of life, with a decrease in breastfeeding and an increase in the consumption of unhealthy and ultra‐processed foods. Virtually all the studied children consumed sweetened beverages and sugar. The increase in the consumption of ultra‐processed foods occurred concomitantly with the increase in the prevalence of days with no consumption of vegetables and fruit. Breastfeeding was an important protective factor against a greater consumption of ultra‐processed food. Thus, the quality of infant feeding practices must be improved with breastfeeding promotion and greater consumption of fresh and minimally processed food.

Nutrition data have improved since 2013 (Heidkamp et al., 2021; Keats et al., 2021), but more action is needed to ensure that global sustainable development goals can be reached and tracked. Our data contribute to the knowledge of a specific vulnerable group, which might have suffered even more during the COVID‐19 crisis, with the double burden of malnutrition. Thus, data shown might help multisectoral double duty actions (World Health Organization, 2017) in northeast Brazilian slums. National nutrition policy and key strategies should consider promoting breastfeeding and increasing fresh and minimally processed food consumption among children under 2 years.

## AUTHOR CONTRIBUTIONS

Laura E. Caulfield, Aldo Ângelo Moreira Lima and Bruna Leal Lima Maciel conducted the research. Aldo Ângelo Moreira Lima and Bruna Leal Lima Maciel designed the study. Eva Débora de Oliveira Andrade, Amanda de Sousa Rebouças, José Q. Filho and Bruna Leal Lima Maciel analysed the data. Eva Débora de Oliveira Andrade and Bruna Leal Lima Maciel wrote the paper. José Q. Filho, Ramya Ambikapathi, Laura E. Caulfield and Aldo Ângelo Moreira Lima critically reviewed the manuscript. All authors have read and approved the final manuscript.

## CONFLICT OF INTEREST

The authors declare no conflict of interest.

## Data Availability

Data are available on request from the authors.
